# RUNX1 induces DNA replication independent of active DNA demethylation at *SPI1* regulatory regions

**DOI:** 10.1186/s12867-017-0087-y

**Published:** 2017-04-04

**Authors:** Shubham Goyal, Takahiro Suzuki, Jing-Ru Li, Shiori Maeda, Mami Kishima, Hajime Nishimura, Yuri Shimizu, Harukazu Suzuki

**Affiliations:** Division of Genomic Technologies, RIKEN Center for Life Science Technologies, 1-7-22 Suehiro-cho, Tsurumi-ku, Yokohama, Kanagawa 230-0045 Japan

**Keywords:** *SPI1*, RUNX1, DNA demethylation, Endogenous expression

## Abstract

**Background:**

SPI1 is an essential transcription factor (TF) for the hematopoietic lineage, in which its expression is tightly controlled through a −17-kb upstream regulatory region and a promoter region. Both regulatory regions are demethylated during hematopoietic development, although how the change of DNA methylation status is performed is still unknown.

**Results:**

We found that the ectopic overexpression of RUNX1 (another key TF in hematopoiesis) in HEK-293T cells induces almost complete DNA demethylation at the −17-kb upstream regulatory region and partial but significant DNA demethylation at the proximal promoter region. This DNA demethylation occurred in mitomycin-C-treated nonproliferating cells at both regulatory regions, suggesting active DNA demethylation. Furthermore, ectopic RUNX1 expression induced significant endogenous *SPI1* expression, although its expression level was much lower than that of natively *SPI1*-expressing monocyte cells.

**Conclusions:**

These results suggest the novel role of RUNX1 as an inducer of DNA demethylation at the *SPI1* regulatory regions, although the mechanism of RUNX1-induced DNA demethylation remains to be explored.

## Background

SPI1 is a hematopoietic lineage-specific TF belonging to the ETS family [1]. It plays an important role in the development of myeloid and lymphoid cells and is highly expressed in monocytes [2, 3]. The expression of *SPI1* is regulated by the combined activity of a proximal promoter and an upstream regulatory element (URE) located −17-kb upstream of the transcription start site (also known as the distal promoter region) in human [3, 4]. Deletion of the URE region contributes to the exacerbation of acute myeloid leukemia (AML) or erythroleukemia [5, 6]. The *SPI1* regulatory regions are differentially methylated in *SPI1*-expressing and nonexpressing cell lines [7, 8]. This differential pattern is also maintained during hematopoietic differentiation, in which ES cells are hypermethylated while hematopoietic stem cells become hypomethylated [7–9]. Meanwhile, abnormal hypermethylation of the *SPI1* regulatory regions is frequently observed in myeloma cell lines with downregulated *SPI1* [10]. Thus, this methylation change seems to be important for *SPI1* expression. However, no molecular mechanism behind the change in DNA methylation status at *SPI1* regulatory regions has been reported yet.

RUNX1 is another TF that is essential for the regulation and maintenance of mammalian hematopoiesis [11]. A previous study reported that RUNX1 regulates *SPI1* at both transcriptional and epigenetic levels [9]. RUNX1 binding at the conserved sites in the URE of *SPI1* is critical for the onset of *SPI1* expression during hematopoietic stem cell formation, making *SPI1* the direct downstream target of RUNX1 [12]. At the pre-hematopoietic or hemangioblast stage, the inception of RUNX1 expression induces chromatin remodeling at the regulatory regions of *SPI1*, in which the binding of RUNX1 to the *SPI1* regulatory region is essential [9]. Moreover, several studies have revealed that chromatin remodeling is coupled with DNA demethylation during embryonic development [13]. Therefore, we hypothesize that RUNX1 may also be involved in recruiting the DNA methylation status change at the *SPI1* regulatory regions.

Here, we describe that the ectopic expression of RUNX1 in HEK-293T cells induces DNA demethylation at the two functionally active regulatory regions of *SPI1* in a replication-independent manner.

## Results

### RUNX1 overexpression induced DNA demethylation at *SPI1* −17-kb URE

**Fig. 1:**
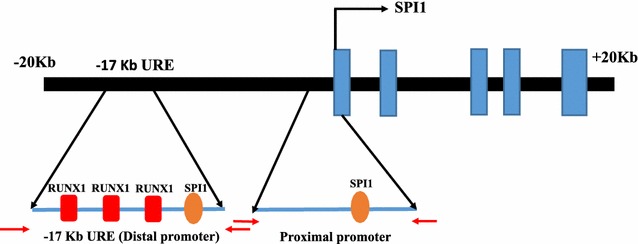
Schematic representation of *SPI1* gene structure: Simplified scheme of the human *SPI1* locus, indicating the two regulatory regions of *SPI1*: distal promoter (−17-kb URE) and proximal promoter. Binding sites for RUNX1 and SPI1 are shown by *red* and *orange boxes*, respectively. The *red horizontal arrows* in the URE and the proximal promoter indicate primers designed for methylation study

**Fig. 2:**
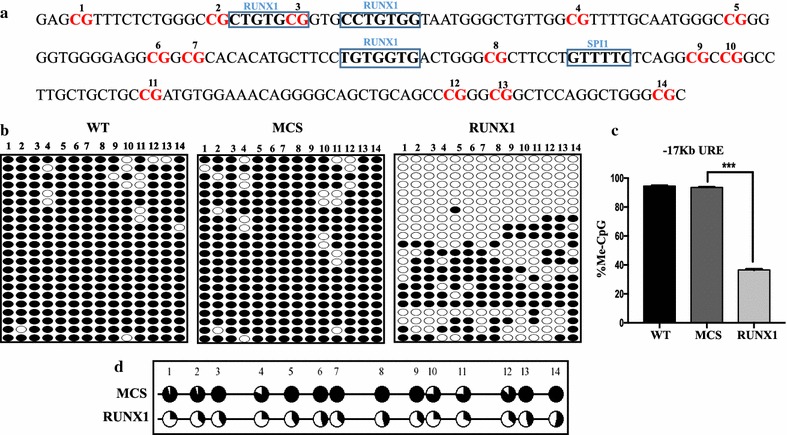
DNA methylation status analysis of *SPI1* −17-kb URE in RUNX1-overexpressing HEK-293T cells: **a** Target sequence of human *SPI1* −17-kb URE. The *number* represents the CpG sites and RUNX1 and SPI1 binding sites are shown in *bold letters*. **b** Methylation pattern diagrams of wild-type, MCS, and RUNX1-overexpressing HEK-293T cells at the *SPI1* −17-kb URE region. *Each row of circles* represents the result of a single amplicon and *each column* represents those of a single CpG site. *Black circles* denote methylated CpGs and *open circles* represent unmethylated ones. The CpG numbers shown correspond to the target nucleotide sequence, shown in **a**. **c** Quantification of percent CpG methylation at the −17-kb URE in wild-type, MCS, and RUNX1-overexpressing HEK-293T cells. The p-value calculated from the comparison between MCS and RUNX1-overexpressing HEK-293T cells was significant (***p < 0.001). **d** Comparison of methylation status graph at each individual CpG site between MCS and RUNX1-overexpressing HEK-293T cells. All of the CpGs were significantly demethylated (p < 0.005)

*SPI1* contains two regulatory regions; one is the −17-kb URE that lies upstream of the transcription start site (also known as the distal promoter region) and the other is the proximal promoter region (Fig. 1). While the −17-kb URE contains three RUNX1 binding sites (Fig. 2a), the proximal promoter region does not contain any such sites [14]. To analyze how RUNX1 expression affects the DNA methylation status of *SPI1* regulatory regions, we first focused on the −17-kb URE. We transduced RUNX1-overexpressing lentivirus into HEK-293T cells, a cell line that does not express RUNX1, followed by DNA methylation analysis by bisulfite sequencing. We observed drastic DNA demethylation in RUNX1-overexpressing HEK-293T cells (Fig. 2b). This DNA demethylation appeared to be RUNX1-specific because we did not see any of these changes in either MCS-transduced (multiple cloning sites: MCS) or wild-type HEK-293T cells. The region as a whole was significantly demethylated (p < 0.001) (Fig. 2c). The DNA demethylation of all individual CpG sites investigated at the −17-kb URE was also found to be significant (p < 0.005) (Fig. 2d).

### RUNX1 overexpression induced partial DNA demethylation at *SPI1* proximal promoter

**Fig. 3:**
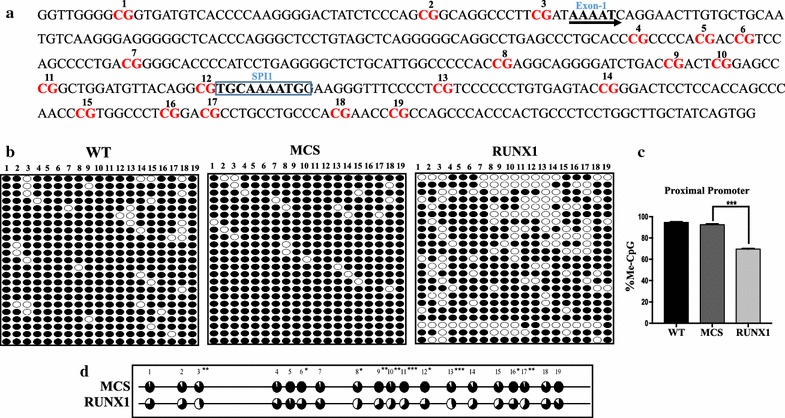
DNA methylation status analysis of *SPI1* proximal promoter in RUNX1-overexpressing HEK-293T cells: **a** Target sequence of the human *SPI1* proximal promoter region. The *number* represents the CpG sites and the SPI1 binding site is also shown. **b** Methylation pattern diagrams of wild-type, MCS, and RUNX1-overexpressing HEK-293T cells at the *SPI1* proximal promoter region. *Each row of circles* represents the result of a single amplicon and *each column* represents a single CpG site. *Black circles* denote methylated CpGs and *open circles* represent unmethylated ones. The CpG numbers shown correspond to the target nucleotide sequence, shown in **a**. **c** Quantification of percent CpG methylation at the proximal promoter region in wild-type, MCS, and RUNX1-overexpressing HEK-293T cells. The p value calculated from the comparison between MCS and RUNX1-overexpressing HEK-293T cells was significant (***p < 0.0001). **d** Comparison of methylation status graph at each individual CpG site between MCS and RUNX1-overexpressing HEK-293T cells. The significantly demethylated CpG sites are marked with *asterisks*, ***p < 0.005, **p < 0.01, *p < 0.05

The proximal promoter region of *SPI1* does not contain any binding site for RUNX1 [14] (Fig. 3a); therefore, we wondered whether RUNX1 can still induce any change in DNA methylation in this region. Our results showed that RUNX1 can induce partial demethylation in the proximal promoter region, with about 30% of CpGs being demethylated (Fig. 3b). Although the demethylation was incomplete, the region as a whole was significantly demethylated (p < 0.0001; Fig. 3c) and the change at most of the CpG sites was also found to be significant (p < 0.005; Fig. 3d), in comparison to that of MCS-transduced cells.

### Molecular mechanism of RUNX1-induced DNA demethylation at *SPI1* regulatory regions

**Fig. 4:**
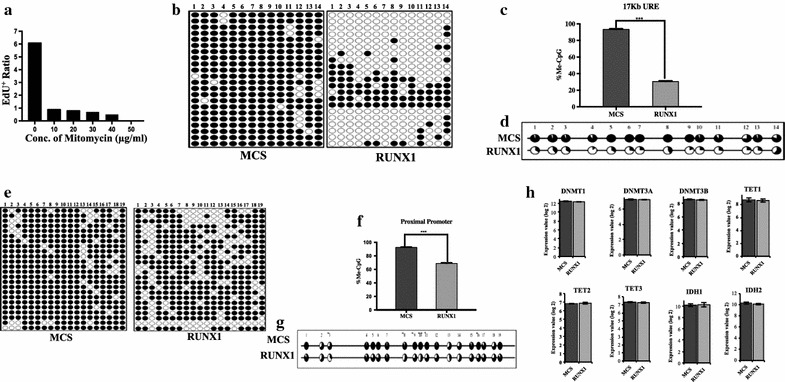
Active DNA demethylation at *SPI1* regulatory regions. **a** Quantification of the concentration of mitomycin-C for cell growth arrest at the G_1_ phase. **b**–**d** Quantification of DNA methylation at the −17-kb URE with 50 µg/ml mitomycin-C treatment. **b** The DNA methylation pattern was measured at the −17-kb URE of MCS- and RUNX1-overexpressing HEK-293T cells. **c** The percentage of methylation in MCS- and RUNX1-overexpressing HEK-293T cells. **d** The methylation difference at each individual CpG site between MCS- and RUNX1-overexpressing HEK-293T cells. All of the sites were significantly demethylated (***p < 0.0001). **e**–**g** Quantification of DNA methylation at the proximal promoter upon 50 µg/ml mitomycin-C treatment. **e** The DNA methylation pattern was measured at the proximal promoter region of MCS- and RUNX1-overexpressing HEK-293T cells. **f** Percentage of methylation in MCS- and RUNX1-overexpressing HEK-293T cells. **g** The methylation difference at each individual CpG site in MCS- and RUNX1-overexpressing HEK-293T cells. All of the sites were significantly demethylated (***p < 0.0001). **h** mRNA expression level of genes involved in DNA methylation and demethylation in MCS- and RUNX1-overexpressing HEK-293T cells (*Expression value* quality control processed signals from microarray data. *Error bar* standard deviation of biological triplicates)

DNA demethylation can be active or passive in nature, where active demethylation occurs by the enzymatic activity of TET enzymes in the absence of replication, while passive demethylation occurs slowly during several rounds of replication [15]. To identify the type of RUNX1-induced demethylation at both *SPI1* regulatory regions, we used mitomycin-C to arrest cell growth at the G_1_ phase. Different concentrations of mitomycin-C were individually tested to identify the concentration when the ratio of Edu positive cells was zero (no DNA synthesis, see “Methods”) (Fig. 4a). We observed drastic DNA demethylation in RUNX1-transduced cells at the *SPI1* −17-kb URE region, even upon mitomycin-C treatment, which would prevent passive DNA demethylation (Fig. 4b). The statistical analysis revealed that the region as a whole was significantly demethylated (p < 0.0001) (Fig. 4c). Significant DNA demethylation was found at each CpG site investigated (Fig. 4d), the same as in the RUNX1-overexpressing cells not treated with mitomycin-C, as shown in Fig. 2d. Interestingly, we also observed RUNX1-induced active DNA demethylation at the proximal promoter region in mitomycin-C-treated cells (Fig. 4e–g). These results demonstrate the ability of RUNX1 to induce active DNA demethylation at both *SPI1* regulatory regions. Recent studies in zebrafish have highlighted that RUNX1 induces the expression of dnmt3bb.1, a DNMT3 paralog in this animal model [16]. Therefore, we next examined whether RUNX1 overexpression enhanced the expression of genes encoding enzymes that are known to be involved in both DNA methylation and demethylation. Specifically, we compared the expression levels of *DNMT1*, *DNMT3A* and *3B*, *TET1-3*, and *IDH1* and *IDH2* between MCS and RUNX1-transduced cells. However, no difference in the expression of these genes was observed (Fig. 4h).

### RUNX1 induces significant *SPI1* expression, but much less than in monocytes

**Fig. 5:**
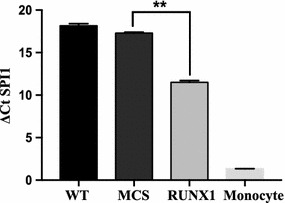
qRT-PCR analysis of endogenous *SPI1* mRNA expression. The endogenous *SPI1* mRNA level was examined in the indicated cell types. Results are presented after normalization to GAPDH, and data are shown as the mean ± SEM (n = 3/cell type). A lower ΔCt value depicts higher expression. The significance represented by the p value was calculated by two-tailed and unpaired Student’s t tests, with the significance levels shown by *asterisks* (**p < 0.005)

DNA methylation at gene regulatory regions generally suppresses gene expression, possibly by blocking transcription factor binding at those regions. To examine whether RUNX1-induced DNA demethylation at *SPI1* regulatory regions can affect *SPI1* endogenous expression, we measured *SPI1* expression in RUNX1-overexpressing HEK-293T cells by qRT-PCR. *SPI1* endogenous expression was significantly (p < 0.005) induced by the RUNX1 ectopic expression, which was estimated to involve a 12-fold upregulation, from the differences in ΔCt values (Fig. 5). However, the *SPI1* expression level for RUNX1-overexpressing HEK-293T cells was still far less than that of monocytes; only 3% of *SPI1* mRNA expression was estimated.

## Discussion

In this study, we found that the ectopic expression of RUNX1 in wild-type HEK-293T cells converts the methylation status of both *SPI1* regulatory regions from hypermethylated to hypomethylated; the results also showed that the induced demethylation was replication-independent active DNA demethylation. Further, RUNX1 overexpression did not change gene expression of enzymes involved in both DNA methylation and demethylation. It has been reported that chromatin remodeling of the *SPI1* URE region in hemangioblasts is induced by the binding of RUNX1, which also accompanies the DNA demethylation of this region [9]. This suggests that RUNX1 binding directly recruits the DNA demethylating machinery. Actually, several TFs have recently shown to be involved in DNA demethylation by recruiting DNA demethylation machinery [17]. On the other hand, the proximal promoter region revealed partial but significant DNA demethylation, although it does not contain any binding sites for RUNX1 and neither any mechanism of its binding is known [12, 14]. Thus, it was interesting to ponder how this replication-independent active DNA demethylation occurs at the proximal promoter region of *SPI1* by the overexpression of RUNX1. Previous chromatin immunoprecipitation (ChIP) data have shed light on the binding of various transcription factors in the URE and proximal promoter regions [8]. Therefore, we speculate that other RUNX1-regulated transcription factor(s) may also be able to induce DNA demethylation at both regulatory regions. Thus, we can conclude that RUNX1 could be directly or indirectly responsible for inducing DNA demethylation at the *SPI1* regulatory regions, although the actual process remains to be confirmed.

Our study also showed that RUNX1 overexpression is not sufficient for inducing the higher level of endogenous *SPI1* expression in HEK-293T cells. The partial demethylation at the proximal promoter region may be responsible for the low level of *SPI1* expression. In fact, the proximal promoter region of *SPI1* is completely hypomethylated in monocytes, in which *SPI1* is expressed at a higher level [18, 19]. Previous reports also suggest the positive correlation between DNA demethylation at gene regulatory regions and gene expression, revealing that active genes are generally hypomethylated while inactive genes are generally hypermethylated at their promoter regions [7, 20]. Furthermore, the cells with high *SPI1* expression regulate this expression by forming an autoregulatory loop between its URE and the proximal promoter region [4, 21], which are in close proximity to each other [22]. Thus, our data suggest that the autoregulatory loop may not form well due to incomplete demethylation at the proximal promoter. The presence of other transcription factors that are expressed in SPI1-expressing hematopoietic cells could also be necessary to induce higher endogenous expression of *SPI1*.

We used HEK-293T cells in this study because they are from a cell line that does not express RUNX1. The propensity of these cells to undergo transfection, their availability, and the possibility of avoiding passage biasness during analysis also make them easy to use. Since our results are derived from nonhematopoietic cells by using an artificial overexpression system, it would be preferable for our results to be evaluated further using hematopoietic cell lines in which RUNX1 and SPI1 are endogenously expressed. However, DNA demethylation at *SPI1* regulatory regions already occurs at the hematopoietic stem cell stage at which RUNX1 is expressed. As the next step, it may be necessary to perform such evaluation in hematopoietic cell lines under specific conditions or with gene manipulation.

## Conclusions

To our knowledge, this is the first study evaluating the potential role of RUNX1 as a demethylating inducer. Our results provide a hint about how demethylation occurs at *SPI1* regulatory regions.

## Methods

### Cell culture and RUNX1 over expression

HEK-293T cells, a human embryonic cell line provided by Riken Cell Bank (RCB), were cultured in High-Glucose Dulbecco’s Modified Eagle Medium (DMEM, Wako, Japan) supplemented with 10% fetal bovine serum (Lonza, Basel, Switzerland) and 2 mM penicillin–streptomycin (Sigma-Aldrich, St. Louis, MO, USA) at 37 °C in 5% CO_2_.

RUNX1 overexpression in HEK-293T cells was carried out by using the lentivirus transduction method. The lentivirus for RUNX1 overexpression and its control MCS were prepared in a mixture of packaging constructs, pCMV-VSV-G-RSV-Rev and pCAG-HIVgp (provided by RIKEN BRC) in accordance with a previously reported protocol [19]. HEK-293T cells (1 × 10^6^ cells per well) were seeded in a poly-d-lysine-coated 12-well plate, followed by transduction on the second day by adding 8 µg/ml polybrene and 10 multiplicity of infection (MOI) of lentivirus in each well. One day after the transduction, the medium was replaced with fresh DMEM medium containing 1 µg/ml puromycin (Thermo Fisher Scientific, Waltham, MA, USA) for the selection of transduced cells. Cells were harvested 7 days after the transduction. Wild-type HEK-293T cells were also kept as a control.

### Bisulfite sequencing

Genomic DNA was isolated from the harvested cells using an All Prep DNA/RNA Mini Kit (Qiagen, Hilden, Germany), in accordance with the manufacturer’s protocol. The DNA quality was assessed by using a Nanodrop 1000 (Thermo Fisher Scientific, Waltham, MA, USA). DNA bisulfite conversion and purification were performed in accordance with the protocol of the EZ DNA Methylation Gold Kit (Zymo Research, Irvine, CA, USA). For amplification of the −17-kb URE (326 bp) and the proximal promoter region (448 bp), previously reported PCR primers [10] were used (Table 1). The amplified PCR products were checked on a 1.5% agarose gel, purified using the PCR purification kit (Qiagen, Hilden, Germany), and then cloned using the Target Clone plus kit (Toyobo, Osaka, Japan). Plasmid DNA was isolated using the QIAprep 96 Turbo miniprep kit (Qiagen, Hilden, Germany) and sequenced in an ABI 3730xl DNA Analyzer. The sequence analysis, including the calculation of methylation percentage, methylation pattern change, and comparison at each CpG site, was performed using the online software QUMA (http://quma.cdb.riken.jp/).

**Table 1:** List of primers used for bisulfite PCR and qRT-PCR

Bisulfite primers	
*SPI1* proximal promoter (Fwd)	GAGATTTTTTGTATGTAGTGTAAGA
*SPI1* proximal promoter (Rv)	TAACTTCCCACTAATAACAAACCA
*SPI1* −17-kb URE (Fwd)	GTTGGATATTTTTTTGAGGTTTGG
*SPI1* −17-kb URE (Rv)	TAAAACCTAAAACTACTAAACCCA
Real-time primers	
GAPDH (Fwd)	GAAATCCCATCACCATCTTCCAGG
GAPDH (Rv)	GAGCCCCAGCCTTCTCCATG
*SPI1* (Fwd)	TCCTGAGGGGCTCTGCATT
*SPI1* (Rv)	TGTCATAGGGCACCAGGTCTT

### Cell cycle arrest

HEK-293T cells (2 × 10^6^ cells per well) were plated into a six-well plate, treated with different concentrations of mitomycin-C (Sigma-Aldrich, St. Louis, MO), and incubated for 4 h at 37 °C in 5% CO_2_. To check cell cycle arrest (at the G_1_ phase), the proliferation assay was performed by following the Click-iT^®^ EdU Cytometry Cell Proliferation Assay protocol (Thermo Fisher Scientific, Waltham, MA, USA); for assessment, FACS was used. After the confirmation of cell cycle arrest at 50 µg/ml mitomycin-C, the cells were plated and transduced with RUNX1 lentivirus on the second day by following the above-mentioned protocol. Wild-type 293T cells were also treated with 50 µg/ml mitomycin-C and transduced with MCS, which were kept as a control.

### DNA microarray

Total RNA was isolated by using the Nucleospin^®^ RNA kit protocol (Macherey–Nagel, Germany). Five hundred nanograms of total RNA was amplified using the Ambion total RNA amplification kit (Ambion, Carlsbad, CA), followed by hybridization of the synthesized cRNA with the Human HT-12 v4 Expression BeadChip kit (Illumina, San Diego, CA, USA), in accordance with the manufacturer’s protocol. Scanning of the chip was performed using Illumina BeadScan and BeadStudio (version 3.1). Data were processed with a package from Bioconductor (lumi) [23, 24] using the free software environment R (http://www.r-project.org/). The microarray data has been registered in gene expression omnibus (https://www.ncbi.nlm.nih.gov/geo/) in NCBI (GSE95308).

### qRT-PCR

Total RNA was isolated using the Nucleospin^®^ RNA kit protocol (Macherey–Nagel, Germany). Reverse transcription of total RNA was performed using the Prime Script RT kit (Takara, Takara Bio, Japan); qRT-PCR was performed with the ABI PRISM^®^ 7500 sequence detection system (Applied Biosystems, USA) using SYBR Premix Ex Taq™ II (Tli RNaseH plus, Takara Bio, Japan) and gene-specific primers for *SPI1* and *GAPDH* (Table 1). PCR cycling conditions consisted of initial denaturation at 95 °C for 10 s, followed by 40 cycles at 95 °C for 5 s and 60 °C for 30 s. Data was analyzed using the 2^−ΔΔCt^ method [25]. Human peripheral blood CD14^+^ monocytes (Lonza, Basel, Switzerland) were kept as a positive control for the comparison of *SPI1* expression.

### Statistical analysis

All of the statistical analyses for DNA methylation status and calculation of methylation percentage were performed using the online quantification tool QUMA. The significance of differences between the transduced and untransduced cells was determined by using unpaired, two-tailed, and Student’s tests (t test), where p < 0.05 was considered significant.

## Abbreviations

TF: transcription factor
URE: upstream regulatory element
AML: acute myeloid leukemia
MCS: multiple cloning sites
ChIP: chromatin immunoprecipitation
MOI: multiplicity of infection
