# Erratum to: RUNX1 induces DNA replication independent active DNA demethylation at *SPI1* regulatory regions

**DOI:** 10.1186/s12867-017-0088-x

**Published:** 2017-04-21

**Authors:** Shubham Goyal, Takahiro Suzuki, Jing‑Ru Li, Shiori Maeda, Mami Kishima, Hajime Nishimura, Yuri Shimizu, Harukazu Suzuki

**Affiliations:** Division of Genomic Technologies, RIKEN Center for Life Science Technologies, 1-7-22 Suehiro-cho, Tsurumi-ku, Yokohama, Kanagawa 230-0045 Japan

## Erratum to: BMC Molecular Biol (2017) 18:9 DOI 10.1186/s12867-017-0087-y

The original article [1] contained an error in the title whereby the word ‘of’ was mistakenly inserted between ‘independent’ and ‘active’ during the article’s production.

As such, the original article title has now been updated to reflect the correct title.

## References

[1] Goyal S, Suzuki T, Li J-R, Maeda S, Kishima M, Nishimura H, Shimizu Y (2017). Suzuki H RUNX1 induces DNA replication independent active DNA demethylation at *SPI1* regulatory regions. BMC Mol Biol.

